# Effect of altitude and harvest year on nutraceutical characteristics of *Rubus ellipticus* fruits

**DOI:** 10.3389/fnut.2024.1419862

**Published:** 2024-09-04

**Authors:** Jyoti Dhatwalia, Amita Kumari, Ishita Guleria, Rakesh Kumar Shukla, Na’il Saleh, Heba A. S. El-Nashar, Mohamed El-Shazly

**Affiliations:** ^1^School of Biological and Environmental Sciences, Faculty of Sciences, Shoolini University, Solan, India; ^2^Department of Botany, Sri Sai University Palampur, Kangra, India; ^3^Patanjali Herbal Research Division, Patanjali Research Foundation, Haridwar, India; ^4^School of Physical and Materials Science, Faculty of Science, Shoolini University, Solan, India; ^5^Department of Chemistry, College of Science, United Arab Emirates University, Al Ain, United Arab Emirates; ^6^Department of Pharmacognosy, Faculty of Pharmacy, Ain Shams University, Cairo, Egypt

**Keywords:** yellow Himalayan raspberry, altitudes, nutritional value, *Rubus ellipticus*, Rosaceae family

## Abstract

*Rubus ellipticus* Smith is an evergreen shrub in the Rosaceae family, commonly known as yellow Himalayan raspberry. The objective of this study is to determine the morphological analysis, minerals, proximate, ascorbic acid, anthocyanins, and carotenoids content in *R. ellipticus* fruits. The fruit samples were collected from four different sites with different altitudes [500 m (District Bilaspur), 1,000 m (District Hamirpur), 1,500 m (District Solan) and 2,000 m (District Shimla)] of Himachal Pradesh for the two consecutive years (2018 and 2019). The fruit morphological investigation revealed that the maximum length (7.71 ± 0.08 mm), width (8.71 ± 0.03 mm), and weight (0.80 ± 0.01 g) of fruits is achieved at higher altitudes (2,000 m) in the year 2018 as compared to 2019. The mineral content (0.05–36.6 mg/g DW), ascorbic acid content (14.59–23.64 mg/g DW), proteins (95.20–131 mg/g DW), and crude fibers (5.6–11.5%) were also higher in fruits grown at 2,000 m altitude, whereas carbohydrates (210–398 mg/g DW), crude fat (2.4–4.1%), and anthocyanins (0.42–1.35 mg/100 g FW) contents were higher in fruits collected from 500 m altitude. According to the results, *R. ellipticus* fruits were rich in crude fiber, moisture, carbohydrates, protein, ash, and crude fat, as well as in micronutrients, and displayed significant variation with altitude in nutrient content. This could be due to the different environmental, geographical, and weather conditions. The high nutrient content of *R. ellipticus* suggests its future potential applications for the food and pharmaceutical industry.

## 1 Introduction

The wild edible fruits and berries have provided nutrients, minerals, and energy to humans since time immemorial and are considered healthy food resources (1, 2). These fruits are also beneficial to health and are used in the pharmaceutical and cosmetic industries for their healing and protective qualities (1, 3–5). In addition, fruits also offer taste and nutrients for a wide range of culinary uses, making them indispensable. Furthermore, studies have shown that wild fruits are higher in nutrients than cultivated fruits (6–8).

Recent advances in analytical technologies and research have confirmed the importance of wild edible fruits as sources of bioactive compounds, making them potential nutraceuticals or functional foods (9, 10). Nutraceuticals are defined as foods that are beneficial to health and help in preventing diseases” (11, 12). Wild fruits contain significant amounts of nutrients as well as biologically active compounds (13–15). Owing to their promising nutraceutical properties, these fruits are valued for their contribution to rural communities’ economic stability, health, and food security in developing nations (7, 16). The World Health Organization recommends eating more than 400 g of fruits and vegetables daily can promote general health and lower the risk of cardiovascular diseases (17, 18).

About 1,532 edible wild species are available in India, and out of these species, 675 species are found in the Himalayan region (19, 20). Among all these plants, *Rubus* is the largest and most diverse genus of the family Rosaceae with almost 750 species (21). *Rubus* fruits are not only beneficial for health but also highly sought after for their delicious flavor and abundant presence of biologically active compounds such as anthocyanins, tannins, phenolics, and flavonoids (22, 23). Members of this genus have been cultivated for centuries for their edible fruits and sometimes they are processed to make products such as jam, wine, tea, ice cream, desserts, seedless jellies, and bakery products. In addition to their nutritional benefits, *Rubus* fruits offer a wide range of culinary possibilities, making them a versatile ingredient in various dishes and beverages (24, 25).

*Rubus ellipticus* Smith, commonly known as yellow Himalayan raspberry, is an important wild plant rich in nutraceuticals. It grows across the subtropical Himalayas (26). It is an evergreen shrub (2.2 m tall), and is generally found near a natural water source, and has a fruit ripening season from the last week of April to the first week of May. It grows at elevations ranging from 300 to 2,600 m and can be found on roadsides, hillsides, mountain valleys, and in sparse forests (27, 28). Yellow Himalayan raspberries are rich in phytochemicals and reported to have various pharmacological properties (29). The paste of young fruits has been used to treat gastritis, diarrhea, and dysentery (26, 30).

In several reports, it has been suggested that the nutrient content of fruits is influenced by a variety of factors, including altitude, harvesting season, location, and environmental factors (31–33). Therefore, the goal of the current study was to investigate the morphological and nutritional characteristics of *R. ellipticus* fruits from different elevations (500, 1,000, 1,500, and 2,000 meters mean above sea level) of Himachal Pradesh to examine the effect of altitude and harvest year on nutrient composition. The experiments were performed during two consecutive years (2018–2019) to study year-to-year variation. The information presented in our study will be valuable in increasing knowledge on the nutritional value of *R. ellipticus*, and factors affecting nutrient concentration, including altitude and year of harvesting, which will help food industries to select the fruits with the optimum composition for their products.

## 2 Materials and methods

### 2.1 Chemicals used

Nitric acid (Hi-Media), perchloric acid (Hi-Media), diethyl ether (Hi-Media), sulphuric acid(Hi-Media), sodium hydroxide (Hi-Media), phosphate buffer (Hi-Media), alkaline copper solution (Hi-Media), Folin–Ciocalteu phenol reagent (Sigma-Aldrich), bovine serum albumin (Hi-Media), hydrochloric acid (Hi-Media), sodium carbonate (Hi-Media), anthrone (Hi-Media), glucose (Hi-Media), trichloroacetic acid (Hi-Media), activated charcoal (Hi-Media), DNPH reagent (Sigma-Aldrich), thiourea (Loba), butylated hydroxytoulene (Hi-Media), ethanol, hexane (Loba), potassium chloride (Hi-Media), sodium acetate (Hi-Media).

### 2.2 Site selection and plants identification

**Figure 1:**
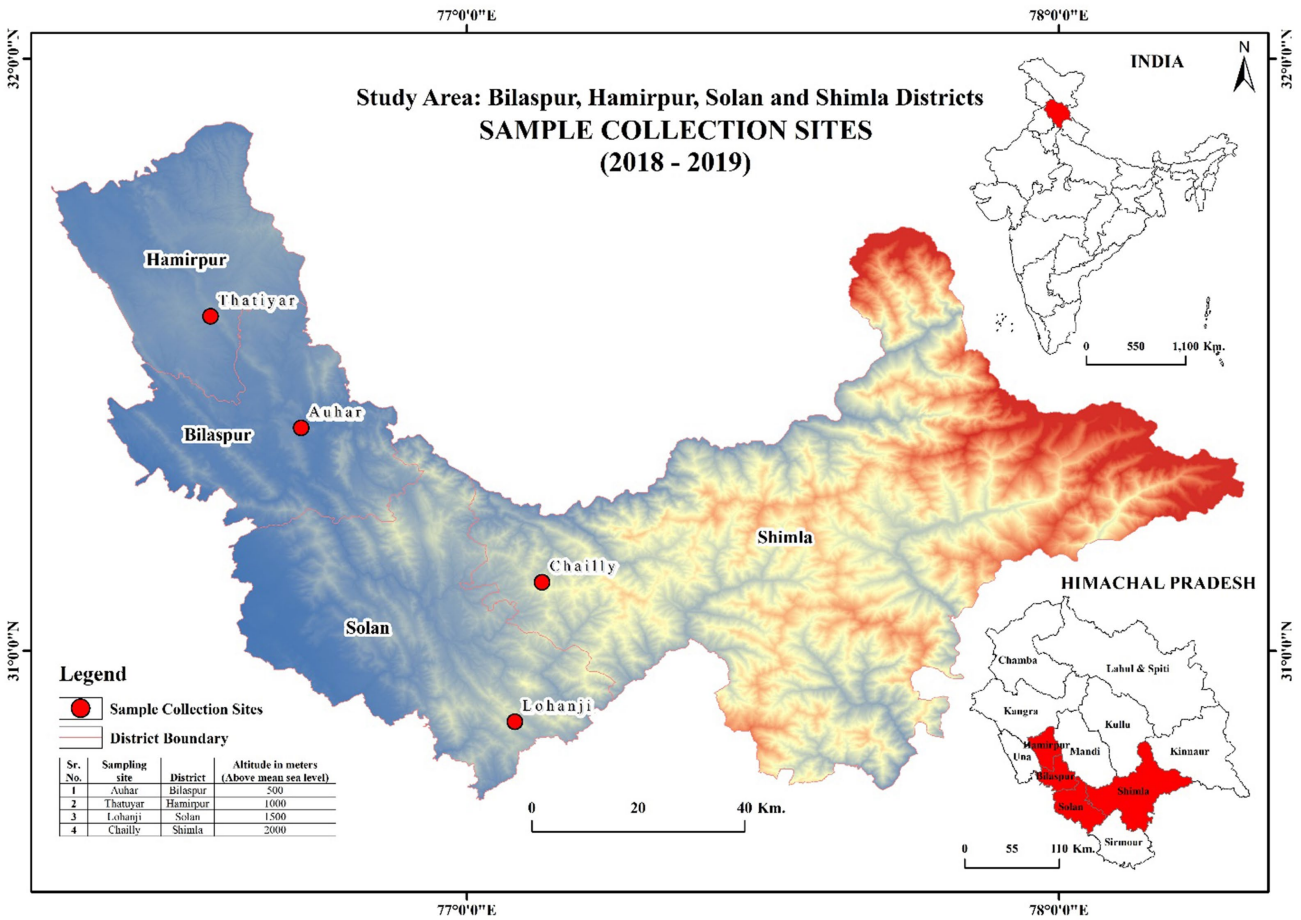
Map of Himachal Pradesh showing sampling sites for two consequent years (2018 and 2019).

Four different sites with four different altitudes [500 m (Auhar, District Bilaspur), 1,000 m (Thatiyar, District Hamirpur), 1,500 m (Lohanji, District Solan), and 2,000 m (Chailly, District Shimla)] in Himachal Pradesh, India, were selected for the selection of *R. ellipticus* plants (Figure 1). *R. ellipticus* was identified in the field based on its morphology (thorny fruiting shrub with trifoliate leaves of the Rosaceae family). Plant twigs were collected from selected plants and used for herbarium preparation. The authentication of the plants was done at the Botanical Survey of India (BSI), Dehradun, Uttarakhand, India, with accession numbers 400–403.

### 2.3 Collection of fruits and their processing

For the sample collection from each altitude, a total of three plants were chosen (based on morphology). The months of March through June mark the beginning of the fruiting season, and April and May mark the beginning of the maturing of fruits, which turn green, into yellow or orange. After ripening (yellow orange-coloured fruits), 300 g of fresh fruits (100 g from each plant) were collected from the selected plants in April and May of the subsequent 2 years (2018 and 2019). The fruit sample from each altitude was collected separately in triplicate and kept in polyethylene bags, and an icebox for further experiments. Furthermore, for homogeneity in sampling of 2018 and 2019, the same plants were selected for the sample collection from each altitude (3 plants per altitude and 100 g per plant) in both years. Additionally, the sample size in the current study consists of 12 samples from 2018 and 12 samples from 2019 (three plants per altitude).

After sample collection, the collected fruits were brought to the laboratory and washed with tap water followed by distilled water (dH_2_O) for the removal of all possible dust particles and half of them were shade-dried at room temperature. After shade drying, the fruits were ground using a mixer grinder (Phillips HL7707/00) and the coarse powder was stored in airtight glass containers for further use. Furthermore, the other half of the fresh fruits were stored at −20°C in a deep freezer until they were used for further analysis.

### 2.4 Morphological analysis of fruits

All morphological parameters [length (mm), width (mm) and weight (g)], of fresh fruits were studied by following the method of Orsanic et al. (34). The size [length (mm) and width (mm)] and weight (g) of the fruits were calculated using a Vernier Caliper and a weighing balance, respectively. The experiment was conducted in triplicate from each altitude.

### 2.5 Mineral analysis

#### 2.5.1 Sample preparation

For mineral analysis, the sample was prepared by following the methodology of Krishnamurthy and Sarla (35). The 1 g of dried fruit powder was mixed with 30 ml of a diacid mixture (900 ml of nitric acid + 400 ml of perchloric acid) in a conical flask. After digesting the mixture for 1 h on a hotplate, the mixture was kept at room temperature for 10 min to obtain a clear solution. After that, 50 ml of dH_2_O was added to the digested mixture; the mixture was filtered through a Whatman filter paper number 41. After the filtrate was transferred into a volumetric flask, dH_2_O was added to make 100 ml of the filtrate. A yellow-coloured solution appeared that was further used for the determination of macro and micronutrients. The atomic absorption spectrophotometer (AAS; Shimadzu Corporation) and flame photometer (Systonic) were used to determine micronutrients (Zn, Cu, and Fe) and macronutrients (Ca, Mg, and K), respectively. All the analysis was done on the triplicate samples from each altitude.

### 2.6 Proximate analysis

#### 2.6.1 Estimation of moisture content

The moisture content of fresh fruits was determined by following the Ashif and Ullah (36) method. The fresh fruits were taken in a pre-weighed petri dish and completely dried in an oven at 60°C for 12 h. In the next step, the sample was cooled in a desiccator and weighed again. The experiment was performed in triplicate from each altitude. The moisture content was calculated according to the following formula:


$$\mathrm{Moisture}\;\left(\%\right)=\frac{\mathrm{Weight}\;\mathrm{of}\;\mathrm{fresh}\;\mathrm{fruits}-\mathrm{Weight}\;\mathrm{of}\;\mathrm{dried}\;\mathrm{fruits}}{\mathrm{weight}\;\mathrm{of}\;\mathrm{fresh}\;\mathrm{fruits}}\times 100$$

#### 2.6.2 Determination of ash content

The total ash content was determined by following the methodology of Ashif and Ullah (36). The 1 g of dried fruit powder was taken in a pre-weighed crucible and completely dried in an oven at 100°C for 1 h. The sample was heated at 600°C for 5 h in a muffle furnace (Sigma) until white ash was obtained. The crucible was cooled in a desiccator (Generic) and weighed again. The test was performed in triplicate from each altitude. The ash content was determined using the following formula:


$$\mathrm{Ash}\;\left(\%\right)=\frac{\mathrm{Weight}\;\mathrm{of}\;\mathrm{the}\;\mathrm{dried}\;\mathrm{fruit}\;\mathrm{powder}\;\mathrm{after}\;\mathrm{ashing}\;\left(g\right)}{\mathrm{Weight}\;\mathrm{of}\;\mathrm{dried}\;\mathrm{fruit}\;\mathrm{powder}\;\left(g\right)}\;\times 100$$

#### 2.6.3 Estimation of crude fat

For the estimation of crude fat, the Unuofin et al. (37) methodology was used (37). The 5 g of dried fruit powder was extracted with 100 ml of diethyl ether and the mixture was placed in an orbital shaker (Thermo Fisher Scientific) for 24 h. A pre-weighed clean beaker was used to collect the filtrate. After collecting the residue and treating it with 100 ml of diethyl ether, it was shaken for another 24 h, and the filtrate was collected in the same beaker. The filtrate was dried in a water bath at 50°C and the beaker was weighed again. The experiment was performed in triplicate from each altitude. The crude fat content was calculated as:


$$\mathrm{Crude}\;\mathrm{fat}\;\left(\%\right)=\frac{W2-W1}{W}\times 100$$

Here, W is the weight of the dried fruit sample, W_1_ is the weight of the empty beaker and W_2_ is the weight of the empty beaker + filtrate of the sample

#### 2.6.4 Estimation of crude fiber

The Unuofin et al. (37) methodology was used for the estimation of total crude fiber (37). The 2 g of dried fruit powder was digested with 100 ml of sulphuric acid (1.25%) on a hot plate for 30 min, then filtered under pressure. The residue was washed with boiling water four times. The process was repeated with 100 ml of sodium hydroxide (1.25%). The final residue was dried at 100°C, cooled in a desiccator, and weighed (C_1_). Afterward, it was incinerated at 550°C for 5 h, transferred to a desiccator (Generic) to cool, and weighed again. The test was performed in triplicate from each altitude. The percentage (%) of crude fiber was calculated as:


$$\mathrm{Crude}\;\mathrm{fibre}\;\left(\%\right)=\frac{C1-C2}{\;C}\times 100$$

Here, C is the weight of the dried fruit sample, C_1_ is the weight of the crucible with dried residue and C_2_ is the weight of the crucible with ash.

#### 2.6.5 Estimation of protein content

The total protein content in the fruit was determined by following the method of Rana et al. (38) with a few modifications (38). The 500 mg of dried fruit powder was weighed and ground well in a pestle mortar with 10 ml of phosphate buffer solution. The mixture was centrifuged and the supernatant was used to estimate protein levels. The 0.2 ml of supernatant was pipette out and the final volume was made 1 ml by the addition of dH_2_O in a test tube. The 5 ml of alkaline copper solution was added to each test tube including the blank (without supernatant). The solution was mixed well and allowed to stand for 10 min. The 0.5 ml of Folin–Ciocalteu phenol reagent was added to both test tubes, mixed properly, and incubated at room temperature in the dark for 30 min. After incubation, blue colour was developed. The absorbance of the blue-coloured solution was taken at 660 nm using a UV–vis spectrophotometer (Thermo Fisher Scientific). The total protein content was calculated from the linear regression graph line (y = 0.0014x + 0.0965; R^2^ = 0.977) of bovine serum albumin (BSA) and the results are expressed in mg/g. The experiment was performed in triplicate from each altitude.

#### 2.6.6 Estimation of carbohydrate content

The total carbohydrate content in the fruits of *R. ellipticus* was estimated by Rana et al. (38) method (38). The 100 mg of dried fruit powder was taken in a test tube, and hydrolysed by keeping it a boiling water bath for 3 h with 5 ml of hydrochloric acid (2.5 N). As soon as the effervescence stopped, the solution was cooled to room temperature and neutralized with solid sodium carbonate. The final volume of the mixture was made up to 100 ml by adding dH2O and the solution was centrifuged. The supernatant was collected after centrifugation and used for further analysis. The supernatant (1 ml) was mixed with 4 ml of anthrone reagent (200 mg anthrone in 100 ml concentrated sulphuric acid), heated for 8 min at 100°C, and cooled rapidly. The observance of the dark green color was read at 630 nm using a UV–vis spectrophotometer (Thermo Fisher Scientific). The carbohydrate content was calculated from the linear regression graph line (y = 0.0668x + 0.055: R^2^ = 0.976) of glucose and the results are expressed as mg/g DW.

#### 2.6.7 Estimation of ascorbic acid

The method of Desai and Desai (39) was used for the determination of ascorbic acid in *R. ellipticus* fruits (39). The 1 g of dried fruit powder was mixed with 5 ml of trichloroacetic acid (4%) and the final volume was made up to 10 ml with the addition of dH_2_O. The solution was centrifuged for 10 min at 2,000 rpm and the supernatant obtained was treated with activated charcoal and then properly mixed on a cyclomixer (Labman) for 5 min. After this, the charcoal was removed, and an aliquot was used for ascorbic acid estimation. Trichloroacetic acid (4%) was used to make up the final volume of 2 ml from the 1 ml of supernatant. To the solution, 0.5 ml of DNPH (2,4-Dinitrophenylhydrazine) reagent was added, followed by two drops of thiourea solution (10%). The content was mixed well and then placed in a water bath for 3 h at 37°C. The resultant yellow crystals were dissolved in 2.5 ml of sulphuric acid solution (85%). The absorbance of the solutions was taken at 540 nm using a UV–vis spectrophotometer. Ascorbic acid content was calculated from the linear regression graph line (y = 0.011x + 0.0275: R^2^ = 0.939) of standard ascorbic acid and the results are represented as ascorbic acid equivalents (mg/g DW). The test was performed in triplicate from each altitude.

#### 2.6.8 Estimation of total carotenoids and lycopene contents

Total carotenoids and lycopene were calculated using the technique outlined by Pasupuleti and Kulkarni (40), respectively. Fresh fruits (0.6 g) were homogenized with 5 ml of butylated hydroxytoulene (0.05% in acetone), 5 ml of ethanol (95%), and 10 ml of hexane. Following centrifugation, the supernatant was added to 3 ml of water and kept on ice. The tubes were incubated at room temperature for the next 5 min. After that, the upper hexane layer was carefully transferred to another tube. The absorbance of this layer was read at 450 nm for carotenoid estimation and at 503 nm for lycopene estimation. The test was performed in triplicate from each altitude. The lycopene concentration of the sample was determined using the formula 1.0 = 3.1206 μg of lycopene per milliliter (OD). The total carotenoids and lycopene contents were calculated as:


$$\mathrm{Carotenoids}\;\left(\mathrm{mg}/g\;FW\right)=\frac{A\times 4\times Vol.}{\mathrm{Weight}\;\mathrm{of}\;\mathrm{fresh}\;\mathrm{fruits}}\times 100$$

$$\mathrm{Lycopene}\;\left(\mathrm{mg}/g\;FW\right)=\frac{3.12\times A\times Vol.\times 100\;}{\mathrm{Weight}\;\mathrm{of}\;\mathrm{fresh}\;\mathrm{fruits}\times 1000}$$

Here, A—absorbance of the fruit sample, 4 is the conversion factor (used to convert the absorbance value (A450) into the concentration of carotenoids in the sample), 3.12 is a conversion factor for lycopene (used to convert the absorbance value at 503 nm (A503) into the actual concentration of lycopene in the sample) and Vol.—Volume of fruit sample (upper hexane layer).

#### 2.6.9 Estimation of anthocyanin content

The anthocyanin content of the fruit sample was quantified according to the following method (41). The 5 g of fresh fruit pulp was mixed with 50 ml of 80% acidified ethanol (95% ethanol: 1.5 N HCl) and shaken overnight at room temperature. The mixture was then sonicated for 10 min and filtered using a Whatman filter paper number 41. The total anthocyanin content of the fruit pulp was measured by the pH-differential method. Samples were diluted with two different solutions: potassium chloride buffer (0.25 M) and sodium acetate buffer (0.4 M) and pH (pH-1.0 for potassium chloride buffer and pH-4.5 for sodium acetate buffer) was adjusted with concentrated hydrochloric acid. The 5 ml of fruit sample was diluted with 5 ml of buffer solution. The diluted samples were kept for 20 min before measurement. In this study, the absorbance was measured at 520 and 700 nm with dH2O as a blank. The difference in absorbance between the samples with pH-1.0 and pH-4.5 was calculated by using the formula below:


$$A=\left(A520\;\mathrm{nm}-A700\;\mathrm{nm}\right)\;pH-1.0-\left(A520\;\mathrm{nm}-A700\;\mathrm{nm}\right)\;pH-4.5$$

The following equation was used to calculate the monomeric anthocyanin pigment concentration:


$$\mathrm{Monomeric}\;\mathrm{anthocyanin}\;\mathrm{pigment}=\frac{A\;x\;MW\;x\;DF\;x\;1000}{e\times l}$$

Here, MW -molecular weight (449.2) of cyanidin-3-glucoside, e-molar absorptivity (26,900 of cyanidin −3-glucoside), DF—dilution factor, l —path length, and A—absorbance of fruit sample. The results were expressed as mg cyanidin-3-glucoside equivalents/100 g FW. The experiment was performed in triplicate from each altitude.

### 2.7 Data analysis

The data was expressed as mean ± SEM and *p* < 0.05 were considered statistically significant. All the statistical data analysis was carried out by SPSS software using paired sample *t*-tests.

## 3 Results and discussion

### 3.1 Morphological analysis

**Figure 2:**
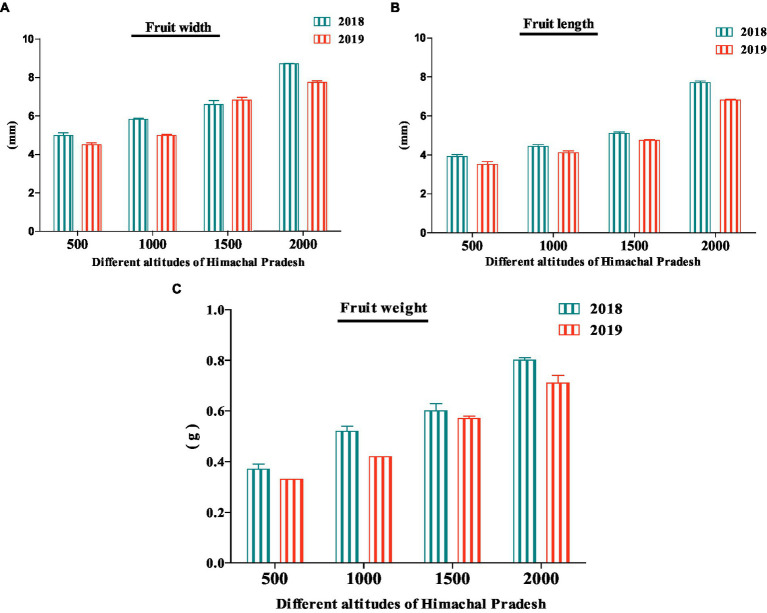
Morphological parameters **(A–C)** of yellow Himalayan raspberries collected from four different altitudes of Himachal Pradesh.

The morphological analysis is a common approach in comparing, identifying, and categorizing botanical samples (42). In the present study, *R. ellipticus* fruits were collected from four different altitudes of Himachal Pradesh, and their size, length, and weight were compared morphologically. The results showed that for both years (2018 and 2019) the fruits collected from 2,000 m altitude had a larger length (2018: 7.71 ± 0.08 mm; 2019: 6.81 ± 0.05 mm), width (2018: 8.71 ± 0.03 mm; 2019: 7.74 ± 0.08 mm), and weight (2018: 0.80 ± 0.01 g; 2019: 0.71 ± 003 g) (Figure 2). Similar to the present study, Ahmed et al. (24) reported the maximum length (9.1 mm), and width (11.4 mm) of *R. idaeus* fruits collected from higher altitudes (1,674–1,981 m) (24). Guerrero-Chavez et al. (43) also observed the higher weight of strawberry (*Fragaria ananassa*) fruits from 1,500 m altitude and minimum from 900 m altitude for the years 2011–2012 (43). The variability in the morphology of fruits could be due to the unique genetic makeup of plant species and environmental conditions such as water deficit and temperature (42). According to Murray et al. (44) and Naizaque et al. (45), the larger fruit weight at higher altitudes could be due to a higher transpiration rate related to higher radiation which would provide a prolonged influx of water and nutrients to the fruits.

The results showed significant (*p* < 0.05) altitudinal variations within the contents of Fe, Ca, K, and Mg in 2018 with few exceptions except Cu and Zn contents, whereas 2019 data showed almost significant (*p* < 0.05) variation (Table 1). Between the years 2018 and 2019, the results showed significant (*p* < 0.05) variation for Cu, K, and Mg contents and non-significant (*p* > 0.05) variation for Ca, Fe, and Zn contents (Table 1).

**Table 1:** Paired sample *t*-test analysis of mineral content of *R. ellipticus* fruits within the altitudinal sites and between years 2018 and 2019.

Sampling sites	Cu	Zn	Fe	Ca	K	Mg
2018						
500 vs. 1,000 m	ns	ns	*	ns	*	*
500 vs. 1,500 m	ns	ns	*	*	*	*
500 vs. 2,000 m	ns	ns	*	*	*	*
1,000 vs. 1,500 m	ns	ns	ns	*	*	*
1,000 vs. 2,000 m	ns	ns	*	*	*	*
1,500 vs. 2,000 m	ns	ns	*	*	*	*
2019						
500 vs. 1,000 m	*	*	*	*	ns	ns
500 vs. 1,500 m	*	*	*	*	*	*
500 vs. 2,000 m	*	*	*	*	*	*
1,000 vs. 1,500 m	ns	*	*	*	ns	ns
1,000 vs. 2,000 m	*	*	*	ns	ns	ns
1,500 vs. 2,000 m	*	*	*	*	*	ns
2018–2019	*	ns	ns	ns	*	*

*Significant (*p* < 0.05), ns, non-significant (*p* > 0.05).

The paired sample *t*-test showed significant (*p* < 0.05) variation w.r.t. fruit length and fruit weight between both years and also within altitudes among various sites (Figure 2; Table 2). Whereas fruit width data showed non-significant (*p* > 0.05) variation between both years’ data and significant variation within altitudes among various sites (Table 2).

**Table 2:** Paired sample *t*-test analysis of morphological parameters of *R. ellipticus* fruits within the altitudinal sites and between years 2018 and 2019.

Sampling site	Fruit weight	Fruit length	Fruit width
2018			
500 vs. 1,000 m	*	*	*
500 vs. 1,500 m	*	*	*
500 vs. 2,000 m	*	*	*
1,000 vs. 1,500 m	ns	*	*
1,000 vs. 2,000 m	*	*	*
1,500 vs. 2,000 m	*	*	*
2019			
500 vs. 1,000 m	*	*	*
500 vs. 1,500 m	*	*	*
500 vs. 2,000 m	*	*	*
1,000 vs. 1,500 m	ns	*	*
1,000 vs. 2,000 m	*	*	*
1,500 vs. 2,000 m	ns	*	*
2018 vs. 2019	*	*	ns

*Significant (*p* < 0.05), ns, non-significant (*p* > 0.05).

### 3.2 Mineral content

Micronutrients and macronutrients are essential for plants to complete their life cycle (46). The results of mineral (macro and micronutrients) analysis showed that for both years (2018 and 2019), the maximum Mg (2018: 0.43 ± 0.00 mg/g DW; 2019: 0.19 ± 0.00 mg/g DW), Zn (2018: 0.14 ± 0.04 mg/g DW; 2019: 0.14 ± 0.00 mg/g DW), Fe (2018: 0.17 ± 0.00 mg/g DW; 2019: 0.28 ± 0.01 mg/g DW), and Cu (2018: 0.13 ± 0.02 mg/g DW; 2019: 0.09 ± 0.00 mg/g DW), contents were observed from the fruits collected from higher altitude (2,000 m) and the minimum from lower altitude (500 m) (Table 3). Whereas K (2018: 36.64 ± 0.41 mg/g DW) and Ca (2018: 9.98 ± 0.19 mg/g DW; 2019: 10.10 ± 0.30 mg/g DW) contents were observed higher in the sample collected from 1,500 m altitude and lower from 500 m altitude. In all sites, K content was the highest followed by Ca, Mg, Fe, Zn, and Cu (Table 3). In a previous study, Ahmad et al. (47) reported 15, 8.6, 6.2, and 0.175 mg/g of Na, K, Ca, and Zn, respectively, in *R. ellipticus* fruits (47). Andola and Purohit (48) and Kumar et al. (49) reported higher mineral contents in the fruits of *Spondias pinnata* and *Malus domestica*, respectively grown at a higher elevation (48, 49). According to Ruiz-Rodriguez et al. (50) and Nyanga et al. (51), the macronutrients and micronutrients of fruits can vary from year to year (50, 51). The fruits’ mineral content not only depends on the species or varieties but also on environmental conditions such as light exposure, temperature, water supply, and weather conditions (31–33).

**Table 3:** The mineral content of *R. ellipticus* fruits.

Altitudes (Mean above sea level)	Cu (mg/g DW DW)	Zn (mg/g DW DW)	Fe (mg/g DW DW)	Ca (mg/g DW DW)	K (mg/g DW DW)	Mg (mg/g DW DW)
2018						
500 m	0.09 ± 0.01	0.08 ± 0.02	0.11 ± 0.00	9.50 ± 0.05	23.70 ± 0.50	0.42 ± 0.00
1,000 m	0.10 ± 0.01	0.10 ± 0.01	0.14 ± 0.00	9.66 ± 0.12	31.50 ± 0.39	0.42 ± 0.00
1,500 m	0.11 ± 0.01	0.10 ± 0.01	0.15 ± 0.01	9.98 ± 0.19	36.64 ± 0.41	0.42 ± 0.00
2,000 m	0.13 ± 0.02	0.14 ± 0.04	0.17 ± 0.00	8.70 ± 0.20	27.73 ± 0.17	0.43 ± 0.00
2019						
500 m	0.05 ± 0.00	0.12 ± 0.00	0.07 ± 0.00	7.40 ± 0.13	10.86 ± 0.56	0.14 ± 0.02
1,000 m	0.06 ± 0.00	0.12 ± 0.00	0.10 ± 0.00	8.17 ± 0.58	12.33 ± 1.02	0.17 ± 0.00
1,500 m	0.07 ± 0.00	0.13 ± 0.01	0.19 ± 0.02	10.10 ± 0.30	12.06 ± 0.45	0.18 ± 0.00
2,000 m	0.09 ± 0.00	0.14 ± 0.00	0.28 ± 0.01	8.32 ± 0.07	14.86 ± 0.25	0.19 ± 0.00

Values are expressed as mean ± standard error mean.

### 3.3 Proximate composition

**Figure 3:**
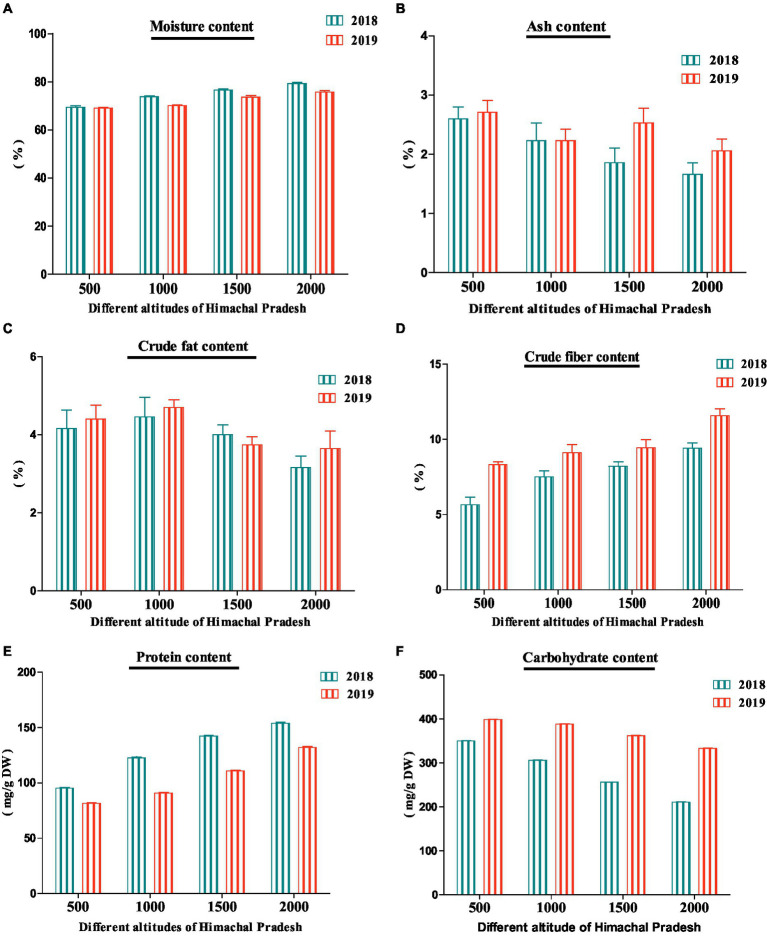
Proximate analysis (**A**—Moisture content; **B**—Ash content; **C**—Crude fat content; **D**—Crude fibre content; **E**—Protein content; **F**—Carbohydrate content) of *R. ellipticus* fruits collected from four different altitudes of Himachal Pradesh.

The proximate analysis deals with the determination of moisture, carbohydrates, proteins, crude fat, crude fiber, and ash contents (52). The present study observed a significant difference (*p* < 0.05) between the proximate content of *R. ellipticus* fruits analyzed during the years 2018 and 2019 from four different altitudes (Figure 3). The study showed the maximum (2018: 79.16 ± 0.51%; 2019: 75.70 ± 0.70%) moisture content in the fruits collected from 2,000 m for both years whereas a minimum content was observed in the fruits collected from lower altitudes (500 m). A significant difference (*p* < 0.05) was observed between the years and within the altitudes through paired sample *t*-test analysis (Table 4). In previous studies, Saklani et al. (53) and Ahmad et al. (47) reported 64.4 and 66.36% of moisture content in *R. ellipticus* fruits, respectively (47, 54).

**Table 4:** Paired sample *t*-test analysis of proximate content of *R. ellipticus* fruits within the altitudinal sites and between years 2018 and 2019.

Sampling sites	Moisture	Ash	Crude fiber	Crude fat	Protein	Ascorbic acid	Carbohydrates	Carotenoids	Lycopene	Anthocyanin
2018										
500 vs. 1,000 m	*	ns	*	ns	*	*	*	ns	ns	*
500 vs. 1,500 m	*	ns	*	ns	*	*	*	*	ns	*
500 vs. 2,000 m	*	*	*	*	*	*	*	*	ns	*
1,000 vs. 1,500 m	*	ns	*	ns	*	ns	*	*	ns	*
1,000 vs. 2,000 m	*	ns	*	ns	*	*	*	ns	*	*
1,500 vs. 2,000 m	*	ns	*	*	*	*	*	ns	ns	*
2019										
500 vs. 1,000 m	*	ns	ns	ns	*	*	*	ns	ns	*
500 vs. 1,500 m	*	ns	*	ns	*	*	*	ns	ns	*
500 vs. 2,000 m	*	*	*	*	*	*	*	*	ns	*
1,000 vs. 1,500 m	*	*	ns	*	*	ns	*	ns	ns	*
1,000 vs. 2,000 m	*	ns	*	*	*	*	*	ns	ns	*
1,500 vs. 2,000 m	*	ns	ns	*	*	ns	*	ns	ns	*
2018–2019	*	*	*	ns	*	*	*	*	*	ns

*Significant (*p* < 0.05), ns, non-significant (*p* > 0.05).

The maximum ash content (2018: 2.60 ± 0.10%; 2019: 2.71 ± 0.10%) was observed in fruits collected from 500 m altitude and was minimum from fruits at 2,000 m for both years. The results showed both significant (*p* < 0.05) and non-significant (*p* > 0.05) variation within the altitudes whereas between the data of 2 years, the results were almost significant (*p* < 0.05) (Table 4). The present study results were also similar to the results reported by Andola and Purohit (48) and Singh et al. (55) where maximum ash content was observed in the *Spondias pinnata* and *Celtis australis* fruits harvested from lower altitudes, respectively (48, 55). The significant differences (*p* < 0.05) in the moisture content of the fruits collected from different locations and years could be due to water availability, sunlight, and wind exposition, which contribute to fruit desiccation (50, 51). According to Ovando-Martinez et al. (56), and Yang et al. (57), the ash content remains inversely proportional to the moisture content as seen in the current study (56, 57).

Carbohydrates (2018: 350.04 ± 0.70 mg/g DW; 2019: 398.80 ± 0.40 mg/g DW) and fat (2018: 4.46 ± 0.50%; 2019: 4.70 ± 0.20%) contents were maximum in fruits collected at 500 m and 1,000 m elevations, respectively (Figure 3). Rana et al. (38) also reported 2.86% and 770 mg/g of crude fat and carbohydrate contents in *R. ellipticus* fruits, respectively (38). The paired sample *t*-test analysis showed a significant (*p* < 0.05) and non-significant (*p* > 0.05) result between the years and within the altitudes (Table 4). The carbohydrate content observed during the present study was higher in the year 2019 as compared with the year 2018 which could be due to the involvement of physiological factors in the ripening process (50). The carbohydrate content in *Cucurbita moschata* fruits was higher at 300 m altitude than at 1,500 m altitude (58). Literature also showed a negative correlation between the contents of carbohydrates and the moisture contents of fruits as observed in this study (50, 59). Similar to the present study, Andola and Purohit (48) also reported a higher content of crude fat in *Spondias pinnata* fruits collected from lower altitudes (48).

For both years, the crude fiber (2018: 9.40 ± 0.36%; 2019: 11.56 ± 0.45%) and crude proteins (2018: 153.67 ± 0.91 mg/g DW; 2019: 131.87 ± 0.90 mg/g DW) were higher in fruit collected from 2,000 m altitude and lower from 500 m altitude (Figure 3). The crude fiber and protein data showed significant (*p* < 0.05) variation between both years and within altitudes among various sites (Table 4). Saklani et al. (53) reported 3.53% of the crude fiber in *R. ellipticus* fruits (54). In another study, Bhutia et al. (60) also recorded 76 mg/g of crude protein content in *R. ellipticus* fruits (60). Similarly, Singh et al. (55), Andola and Purohit (48), and Singh and Todaria (61) reported a higher protein content in the fruits of higher elevation as compared with the fruits of lower elevation (48, 55, 61). Parra-Coronado et al. (62) and Zhaid et al. (63) also reported a positive correlation between the altitudes and fiber content of *Acca sellowiana* and *Cydonia oblonga* fruits (62, 63).

For both years, the maximum ascorbic acid (2018: 23.64 ± 0.28 mg/g DW; 2019: 22.78 ± 0.23 mg/g DW), carotenoids (2018: 1.78 ± 0.11 mg/g FW; 2019: 1.66 ± 0.14 mg/g FW), and lycopene (2018: 0.013 ± 0.001 mg/g FW; 2019: 0.012 ± 0.003 mg/g FW) contents were found in *R. ellipticus* fruits collected from 2,000 m and lower content in fruits at 500 m (Table 5). In ascorbic acid, the significant difference (*p* < 0.05) was observed between the years, whereas among various sites the difference was mostly significant (*p* < 0.05) as shown in Table 4. Both carotenoids and lycopene contents showed non-significant (*p* > 0.05) variation within the altitudes and significant variation between (*p* < 0.05) both years (Table 4).

**Table 5:** Ascorbic acid, carotenoids, lycopene, and anthocyanin contents in the fruits of *R. ellipticus* collected from different altitudes.

Altitudes (mean above sea level)	Ascorbic acid (mg/g DW)	Carotenoids (mg/g FW)	Lycopene’s (mg/g FW)	Anthocyanin’s (mg/100 g FW)
2018				
500 m	17.83 ± 0.35	1.21 ± 0.20	0.010 ± 0.01	1.17 ± 0.20
1,000 m	21.53 ± 0.34	1.45 ± 0.12	0.011 ± 0.00	0.80 ± 0.10
1,500 m	22.67 ± 0.42	1.59 ± 0.17	0.012 ± 0.00	0.49 ± 0.14
2,000 m	23.64 ± 0.28	1.78 ± 0.11	0.013 ± 0.00	0.44 ± 0.13
2019				
500 m	14.59 ± 0.64	0.89 ± 0.24	0.007 ± 0.00	1.35 ± 0.14
1,000 m	20.84 ± 0.67	1.15 ± 0.12	0.01 ± 0.00	0.79 ± 0.16
1,500 m	21.68 ± 0.29	1.42 ± 0.15	0.010 ± 0.00	0.62 ± 0.14
2,000 m	22.78 ± 0.23	1.66 ± 0.14	0.012 ± 0.00	0.42 ± 0.15

Values are expressed as mean ± standard error mean.

Kumar et al. (49) also reported higher ascorbic acid and carotenoid content (28.80 mg/100 g and 104.50 mg/kg) in *Malus domestica* fruits collected from higher altitudes (1,800 m) (49). The ascorbic acid or vitamin C content of fruits in the present study was higher than those reported by Saklani et al. (53) and Bhusal et al. (64) i.e., 10.5 and 0.19 mg/g, respectively (54, 64). Bhutia et al. (60) observed 0.11 mg/g of carotenoid content in the fruits of *R. ellipticus* (60). According to Ruiz-Rodríguez et al. (50) and Guerrero-Chavez et al. (43), the ascorbic acid and carotenoid contents in fruits are more influenced by the location and year of harvest as well (43, 50).

The lower amount of anthocyanins was found in fruits collected from high altitudes (2,000 m) and higher in low altitudes (500 m) for both years (Table 5). These results showed mostly significant (*p* < 0.05) and non-significant (*p* > 0.05) variations within the altitudes and between the years 2018 and 2019, respectively (Table 4). Bhutia et al. (60) and Badhani et al. (65) reported 3.8 mg/100 g fw and 0.12 mg/100 g fw of anthocyanin content in *R. ellipticus* fruits, respectively (60, 65). Similar to the present study, Rieger et al. (66), Mphahlele et al. (67), and Guerrero-Chavez et al. (43) observed low anthocyanin content in fruits of *Vaccinium myrtillus*, *Punica granatum,* and strawberry at higher altitudes (68, 69). Additionally, other environmental factors such as rainfall, climate, light intensity, maturity stage, and storage also influenced the content of ascorbic acid and carotenoids in fruits (51, 63, 68, 69).

The study revealed that fruits grown at higher altitudes had the highest mineral and nutrient content compared to those grown at lower altitudes. The unique environmental conditions at higher altitudes, such as cooler temperatures and increased sunlight exposure, can result in fruits with a more concentrated flavor profile, may also become valuable for the production of jams and candies. Due to their shorter shelf life, they can be processed and preserved into delicious and nutritious products that can be enjoyed for a longer period of time. Furthermore, it is also recommended to prioritize the cultivation and consumption of fruits grown at higher altitudes in order to harness their superior nutritional benefits and promote overall health and well-being.

## 4 Conclusion

The present research determined the morphological, nutritive, and mineral composition of *R. ellipticus* fruit collected over 2 years (2018 and 2019) at various elevations (500–2,000 m) in Himachal Pradesh. The findings of the current investigation showed that *R. ellipticus* fruits are rich in nutrients and minerals. For both years, the fruits harvested at a higher altitude (2,000 m) had significantly greater amounts of minerals, ascorbic acid, proteins, and crude fibers on the other hand fruits at lower elevations had the largest amount of anthocyanins, crude fat, and carbohydrates. Furthermore, the maximum fruit length, weight, and breadth were reported in fruits harvested at a higher altitude (2,000 m). This might be due to differences in geographical and environmental variables. The results of the current study indicate that altitude has a substantial impact on the nutritional value and physical characteristics of fruits.

*Rubus* fruits are considered a valuable source of daily food for the local population to prevent several nutritional deficiencies and play a significant role in maintaining and regulating metabolic activity. *Rubus* fruits might be utilized to make a variety of food items for customers in the future due to their great nutritional content. In addition to maximizing their nutritional benefits, cultivating these wild fruit species can also open up opportunities for future research and development in the field of food and medicine. *Rubus* fruits may possess unique compounds and properties that can be utilized in various applications, further enhancing their value and potential impact.

## Data Availability

The original contributions presented in the study are included in the article/supplementary material, further inquiries can be directed to the corresponding authors.
